# Ischemic Stroke in Women: Understanding Sex-Specific Risk Factors, Treatment Considerations, and Outcomes

**DOI:** 10.3390/jcdd11120382

**Published:** 2024-11-29

**Authors:** Pei Chia Eng, Lyeann Li Ying Tan, Tamara N. Kimball, Savvina Prapiadou, Benjamin Y. Q. Tan

**Affiliations:** 1 Division of Endocrinology, Department of Medicine, National University Hospital, Singapore 119074, Singapore; p.eng@nus.edu.sg (P.C.E.); lyeann.tan@gmail.com (L.L.Y.T.); 2 Department of Metabolism, Digestion and Reproduction, Imperial College London, London W12 0NN, UK; 3 Department of Neurology, Massachusetts General Hospital, Boston, MA 02114, USA; tkimballdesantiago@bwh.harvard.edu (T.N.K.); sprapiadou@mgh.harvard.edu (S.P.); 4 Broad Institute of MIT and Harvard, Cambridge, MA 02142, USA; 5 Division of Neurology, Department of Medicine, National University Hospital, Singapore 119074, Singapore

**Keywords:** ishemic stroke, women, gender, risk factors, menopause, estrogens

## Abstract

Ischemic stroke is a major cause of mortality and disability and has become a significant public health concern among women. Overall, women have more ischemic stroke events than men, in part due to their longer life span, and also suffer from more severe stroke-related disabilities compared to men. Women are also more likely than men to present with atypical non-focal neurological symptoms, potentially leading to delayed diagnosis and treatment. Female-specific risk factors, especially those related to pregnancy, are often under-recognized. A woman’s risk for ischemic stroke evolves throughout her lifespan, influenced by various factors including the age of menarche, pregnancy and its complications (such as parity, pre-eclampsia/eclampsia, and preterm delivery), postpartum challenges, oral contraceptive use, and menopause. Additionally, vascular risk factors like hypertension, diabetes, and atrial fibrillation are more prevalent among older women. Despite comparable treatment efficacies, women generally experience poorer outcomes after stroke. They also face higher rates of post-stroke depression, further complicating recovery. Although significant strides have been made in reducing the incidence of ischemic stroke, our understanding of the unique risks, underlying causes, and long-term consequences for women remains limited. While sex hormones may explain some differences, a lack of awareness regarding sex-related disparities can result in suboptimal care. This review aims to illuminate the unique risks and burdens of ischemic stroke faced by women, advocating for a more nuanced understanding to enhance prevention and treatment strategies.

## 1. Introduction

Stroke is a major cause of death in women and is increasingly being recognized as a cause for public health concern in women [1]. Although the prevalence of stroke has been reported to be higher in men than in women, this prevalence rate reverses after menopausal age with more women suffering from stroke than men [2,3]. Women, compared to men, are also more likely to suffer from disability and institutionalization following stroke [2]. Furthermore, due to their longer life expectancy, women are more likely to be widowed at the time of stroke, and social isolation often hinders their ability to achieve full recovery [2]. Indeed, stroke-related disability and mortality rates are higher in women than in men, even after adjusting for pre-stroke functional status [4,5]. Disparities of stroke prevalence have also been reported in different ethnicities. In the Northern Manhattan Stroke Study (NOMAS), women of Black and Hispanic origin had a higher risk of stroke compared to age and sociodemographically matched women of white ethnicity [6]. As the population ages, the disparity in stroke prevalence and outcomes among women is projected to increase significantly. This underscores the urgent need to better understand sex-related stroke disabilities and develop effective, gender-specific strategies for prevention, rehabilitation, and secondary prevention in women [7]. In this review, we explore sex-specific differences in the biology and risks of ischemic stroke (IS). We examine the distinct risk factors and unique burdens faced by women, laying the groundwork for future research and the development of personalized strategies in stroke care.

## 2. Ischemic Stroke Risk Across a Woman’s Lifespan

The lifetime risk of IS in women and men varies according to the geographic location [8]. Globally, due to the longer life expectancy in women, the lifetime risk of IS from age 25 onwards is higher in women than in men [8]. According to the Framingham study, a women’s lifetime risk of stroke is estimated to be 1 in 5 (20 to 21%) compared to 1 in 6 (14% to 17% in men) [9]. Recent data from Canada reveal sex-based differences in the risk of IS and transient ischemic attack across the lifespan. Women under 30 face a higher risk compared to men, while men have a greater risk during midlife. By age 80, the risk becomes equal for both genders [10]. For women, the risk of IS fluctuates at different life stages, notably during pregnancy, postpartum, post menopause, and older age. In addition to traditional vascular risk factors, women face unique risks such as lifetime exposure to estrogen (timing of menarche and menopause), pregnancy-related complications, and menopause itself.

## 3. Traditional Vascular Risk Factors for Ischemic Stroke in Women

Traditional vascular risk factors associated with IS include hypertension, diabetes mellitus, and atrial fibrillation. Hypertension has a direct and a linear relationship with the risk of IS [11]. Notably, blood pressure trajectories rise more sharply in women as they age compared to men, with differences becoming evident as early as the third decade of life [9,12]. Stage 2 hypertension was found to be associated with a 30% higher risk of stroke in women compared to men [13]. Although the underlying biological mechanisms contributing to sex differences in hypertension are not fully understood, hormonal variations throughout a woman’s life, combined with socioeconomic and environmental factors, likely play a role. For example, African American women exhibit a higher prevalence of hypertension and face more challenges in achieving blood pressure control compared to women of white ethnicity [14]. Treatment response rates for hypertension vary amongst men and women. One study found that less than half of individuals with hypertension achieved adequate blood pressure control, with a higher percentage in women compared to men (23% for women versus 18% for men) [15].

Diabetes mellitus, especially type 1 diabetes, was associated with a higher hazard ratio of IS in women than men [13]. In a study evaluating participants from the UK Biobank, diabetes was found to be associated with a higher hazard ratio of IS in women than men (RHR 1.25 [1.00–1.56]) [13]. A meta-analysis of individual participant data from 980,793 adults revealed that even after adjusting for other risk factors, diabetes triples the risk of occlusive vascular mortality in women (RR 3.00, 95% CI 2.71–3.33), while in men, the risk is doubled (RR 2.10, 95% CI 1.97–2.24) [16]. In this same study, the excess absolute risk of mortality in women aged 35–59 was 0.05% (95% CI 0.03–0.07) per year, compared to 0.08% (95% CI 0.05–0.10) per year in men. At ages 70–89, the annual excess risk was 1.08% (95% CI 0.84–1.32) in women, versus 0.91% (95% CI 0.77–1.05) in men [16]. In a meta-analysis involving more than eight million women, women with a history gestational diabetes mellitus were found to have an increased risk of ischemic stroke (risk ratio 1.49, 1.29 to 1.71) [17]. The prevalence of diabetes is lower in pre-menopausal women compared to men; however, the risk of developing diabetes increases significantly after menopause [18]. In postmenopausal women, the presence of diabetes further increases the levels of atherogenic lipid fractions and vascular inflammation markers. This suggests that postmenopausal women have the greatest potential for the development of atherothrombotic complications including IS [19].

Atrial fibrillation (AF) is a significant risk factor for IS [6]. Despite a higher incidence of AF in men compared to women, the prevalence of AF increases in women compared to men after the age of 85 years old [20,21]. Additionally, female sex has been identified as a prognostic factor for stroke in AF patients, particularly among those with two or more non-sex-related stroke risk factors, highlighting the gender-specific risk modification [22]. A pooled meta-analysis found that AF was associated with a two-fold higher relative risk for IS and cardiovascular mortality in women compared with men (RR 1.99 (95% CI 1.41–2.71)) [21]. This increased vulnerability may be exacerbated by disparities in treatment, as several studies have suggested that women with AF are often undertreated with anticoagulants compared to men [20,23].

## 4. Female-Specific Risk Factors for Ischemic Stroke

**Figure 1:**
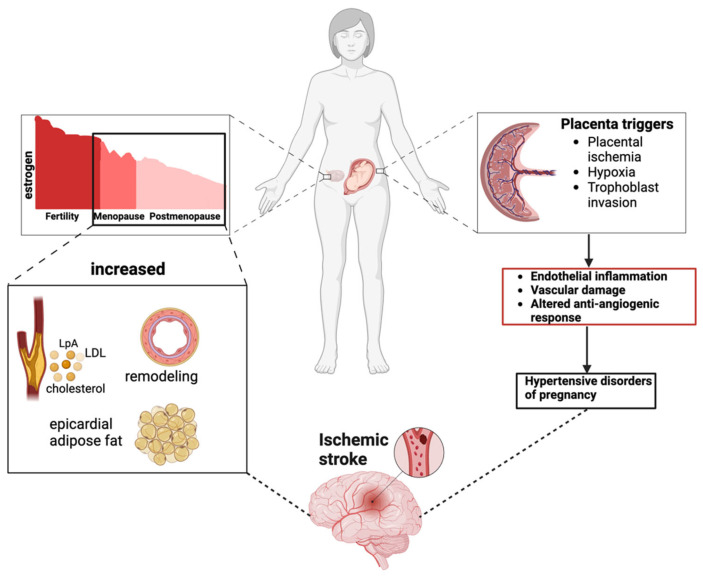
Multifactorial Risk Pathways for Ischemic Stroke in Women. This figure illustrates the interconnected risk factors contributing to ischemic stroke in women, focusing on hormonal variations throughout life, lipid profiles, and pregnancy-related complications.

A myriad of female-specific risk factors contribute to the risk of ischemic stroke. This section explores the interconnected factors influencing ischemic stroke in women, with a focus on hormonal fluctuations across the lifespan, variations in lipid profiles, and pregnancy-related complications as illustrated in Figure 1.

### 4.1. The Role of Estrogen—Insights from Preclinical Studies on Cerebral Vasculature

The role of estrogen and testosterone on cerebral vasculature has been well documented. In vivo studies show that exposure to estrogen or testosterone alters the reactivity of cerebral arteries [24]. Specifically, treatment with estrogen modulates the myogenic tone in cerebral arteries by enhancing the sensitivity to, or the production of vasodilatory factors such as nitric oxide. This modulation occurs likely through a combination of genomic and nongeneomic pathways [24,25]. The estrogen-mediated release of endothelial nitric oxide also appears to decrease leucocyte adhesion in pial venules, thus partially modifying the cerebral inflammatory process [26]. In addition to leucocyte processes, in vivo and in vitro studies showed that estrogen induces additional neuroprotective effects by reducing mitochondria production of free radical species [27]. High estrogen levels also suppress smooth muscle calcium influx which could directly relax cerebral arteries via an estrogen receptor-independent pathway [28]. Intracellular levels of magnesium are also altered following estrogen treatment of female basilar artery smooth muscle cells which could mediate smooth muscle vasodilation [29]. Testosterone treatment of orchiectomized male rats has no effect on nitric oxide levels or nitric oxide mediated dilation in cerebral vessels [25]. Notably, estrogen acts through a number of mechanisms to maintain cerebral autoregulation [24] to partially restore myogenic tone in ovariectomized female rats [30] and low estrogen levels could be a contributory factor in certain cerebrovascular pathologies such as IS risk.

### 4.2. Timing: Age of Menarche and Age of Menopause

In clinical studies, a shorter lifetime estrogen exposure (less than 33 years) has been associated with an increased risk of cardiovascular disease (CVD) [31]. The age at menarche and menopause significantly influences a woman’s total estrogen exposure. Specifically, a U-shaped association with stroke risk has been observed with age at menarche, where both early menarche (≤11 years) and late menarche (≥15 years) are linked to a higher risk of CVD [31]. Similarly, early menopause (age ≤ 40 years) was associated with a 92% higher risk of CVD (HR, 1.92; 95% CI 1.69–2.18) compared to women who experience menopause at the ages of 50 to 51 years [31].

The type of menopause and its timing significantly affect CVD risk [32]. Surgical menopause, which involves an abrupt decline in ovarian hormones, contrasts with the gradual hormonal decline observed in natural menopause. A study analyzing pooled data from 10 international studies involving 203,767 women, found that women who experienced surgical menopause before the age of 39 exhibited a higher risk of CVD compared to women who underwent natural menopause [32]. However, subsequent studies have found no significant association between surgical menopause and stroke risk [33]. Additionally, hormone replacement therapy for women who had surgical menopause before the age of 50 is associated with at least a twofold decrease in the risk of stroke and CVD [32]. Notably, the timing of menopause and menarche are both important factors to consider in assessing risk of CVD for women.

### 4.3. Pregnancy-Related Risk Factors and Post-Partum Changes

The incidence of stroke during pregnancy is reported to be 34.2 per 100,000 deliveries, while the incidence among non-pregnant women of childbearing age is approximately 11 per 100,000 deliveries [34]. Although stroke is considered a rare phenomenon during pregnancy, it causes significant fetal morbidity and mortality and accounts for 12% of maternal death [35]. The risk of stroke is increased by ninefold around the time of delivery and rises up to threefold in the early postpartum period and may persist for up to 12 weeks after delivery [36]. Pregnancy is characterized by a hypercoagulable state caused by imbalance between procoagulant and anticoagulant activity [37]. Fibrinolytic activity is also reduced during pregnancy due to the higher levels of placenta-derived plasminogen activator inhibitor type 2 [37]. Hypercoagulability during pregnancy could cause cerebral venous thrombosis and consequently venous infarcts [37]. Women with pre-existing hematological disease, such as antiphospholipid syndrome and sickle cell disease, have an even higher risk of thrombotic events [35].

Hypertensive disorders of pregnancy (HDP) include gestational hypertension, chronic hypertension and preeclampsia/eclampsia, affecting 116 per 100,000 women of childbearing age [38]. The prevalence of HDP varies depending on the region, with the highest prevalence in Africa, followed by Southeast Asia and the Middle East [39]. Approximately 10 to 25% of women with gestational hypertension will develop pre-eclampsia [39]. In the Stroke Prevention in Young Women Study (aged 15 to 44 years), women with pre-eclampsia were 60% more likely to have a nonpregnancy related IS compared to controls (OR 1.63; 95% CI: 1.02–2.62) [40]. The risk of premature CVD is also elevated in women with maternal placental syndromes, such as gestational hypertension, preeclampsia, eclampsia, and placental abruption [41]. Although many theories have been proposed, the exact mechanism of pre-eclampsia causing CVD is not known. Most theories converge on endothelial inflammation, vascular damage and altered anti-angiogenic response such as overexpression of circulating sFLT-1 in women with a history of pre-eclampsia compared to those without pre-eclampsia [42].

Pregnancy is associated with an increased risk of cerebral angiopathy. This umbrella term encompasses reversible cerebral vasoconstriction syndrome (RCVS) and posterior reversible encephalopathy syndrome of medium and large-sized cerebral arteries. Endothelial dysfunction is thought to be the mechanism that underlies RCVS that occurs during and after pregnancy. Preclinical studies showed that cerebral arteries in late pregnant and postpartum animals dilate at lower pressures compared to nonpregnant animals [43]. Furthermore, cerebral arteries from animals which are postpartum or at late stage of pregnancy exhibit differential response to serotonin compared to non-pregnant animals [43]. The endothelial dilation to serotonin could be related to altered calcium signalling in the postpartum state or the differences in composition of serotonin receptors between nonpregnant and post-partum states [43]. A case–control study found that brachial artery flow mediated endothelium dependent dilatation was lower in women with pre-eclampsia compared to controls after adjusting for cardiovascular risk factors [44]. The changes in vascular and inflammation could partly explain the increased stroke risk in women later in life. Studies on gestational effects on endothelium-dependent vasodilator production in cerebral circulation is needed to understand the mechanisms of RCVS.

### 4.4. Oral Contraceptives

Early studies showed that oral contraceptive (OC) users had a 3-fold increased risk of venous thrombosis compared to nonusers [45,46]. More recent studies reported a low absolute annual absolute risk of 2 to 3 per 10,000 users [47]. Over time, the dose of estrogen in OC preparation have also reduced (from 100 to 150 µg of ethinyl estradiol to 20 to 30 µg). It is unclear if reducing the dose of estrogen could result in lower risk of thrombosis. Some studies showed a lower risk of thrombosis with lower estrogen dose OC but difference in thrombosis rate was not observed in either the 50 or 30 µg ethinyl estradiol users [48,49]. Progesterone content in the different generations of OC preparations have also changed over the years. A meta-analysis reported a 1.7-fold increased thrombosis risk in third generation OC (e.g., desogestrel, gestodene) compared with second-generation OC (e.g., levonorgestrel, norgestrel) [50]. The risk of thrombosis with third-generation OC was higher in the early phases of use compared to prolonged use [50]. Plasma concentration of women on third-generation OC were also found to be tilted towards a prothrombotic state [51]. Overall, OC is associated with an immediate increased risk of stroke within the first year of use in first-time users but not in previous users [52], and the risk is higher in third-generation OC [47]. The lifetime risk of stroke is also not different between those exposed to OC and those who have not been [52].

### 4.5. Menopause

Menopause is a natural life stage characterized by cessation of ovarian function and reduction in ovarian estradiol levels, coupled with a rise in serum follicle stimulating hormone level (FSH) above normal. Cerebral hemodynamic studies showed that cerebral reactivity was significantly lower in postmenopausal women when compared to age-matched premenopausal women [53]. In addition to the decline in estradiol level, the late perimenopausal and postmenopausal stage was also associated with an increase in total cholesterol, low density lipoprotein (LDL) and lipoprotein (a) levels [54]. Using data from the SWAN study, repeated carotid scan measured over the menopausal period showed evidence of structural carotid artery remodelling in the late perimenopausal stage compared to early perimenopausal stages independent of aging [53]. Despite evidence suggesting increased CVD risk with menopause, larger clinical trials did not seem to suggest protective benefits of estrogen replacement in postmenopausal women [55,56,57]. A randomized double-blind, placebo-controlled trial of 1 mg estradiol in postmenopausal women showed that 3 years of treatment with estradiol-17β did not reduce risk of death, fatal or nonfatal stroke, or transient ischaemic attack in women [55]. Another randomized trial from the Heart and Estrogen-Progesterin Replacement Study reported no increase in stroke risk in younger women with coronary disease (*n* = 2763, mean age 67 years old) when treated with estradiol regimen (0.625 mg of conjugated equine estrogen plus 2.5 mg medroxyprogesterone per day) over a 4-year period compared to the placebo arm (HR, 1.23; 95% CI, 0.89–1.70) [57]. The Women’s Health Initiative (WHI), which involved 16,608 healthy postmenopausal women, was stopped prematurely because women in the estrogen group, with or without progesterone, had a 31% increase in cardiovascular events and 40% increase in stroke risk over 5.2 years compared to women in placebo groups [56]. Exogenous estrogen was associated with a higher stroke risk (46% increase in stroke risk) in the younger group (age 50 to 59 years old) compared to other age groups, with the risk becoming apparent by the second year on HRT [56]. More recent data documented the use of low-dose transdermal estrogen formulations could effectively treat menopausal symptoms without increasing stroke risk [58]. Furthermore, the timing of estrogen replacement seems to play a role in atherosclerosis as HRT initiated within 3 years after menopause did not affect carotid intima thickness (CIMT) compared to placebo [59]. Similarly, HRT started within 6 years resulted in smaller increase in CVD compared to HRT initiated 10 years or later after menopause [60].

### 4.6. Metabolic Changes with Menopause

Body fat composition significantly influences an individual’s risk of cardiometabolic complications [61]. Gender specific differences in body fat composition have been well described in the literature [62]. Compared to men, pre-menopausal women have a lower percentage of visceral adipose tissues (VAT) (10 to 20% in men vs. 5 to 8% in women) and tend to accrue fat in subcutaneous tissues which protects them from metabolic syndrome [63]. As a woman transitions through menopause, total adiposity increases, fat free mass decreases, and there is a redistribution of fat from SAT to the VAT [62]. In the SWAN study, the mean annual increase in absolute fat mass increases to 0.45 kg per year during the menopausal period compared to 0.25 kg per year before menopause [64]. Lean mass decreases by 0.05 kg per year during menopause [65]. Overall, no detectable changes in weight or BMI are observed at the onset of menopausal transition [65], but changes in body fat composition [63] and cardiovascular fat deposition (characterized as epicardial adipose tissue, para/pericardial adipose tissue and perivascular adipose tissue) could play a role in higher CVD risk in women after menopause [66]. Epicardial adipose tissue (EAT) deposition has been thought to directly impact atherosclerosis due to anatomical proximity but more importantly, EAT secretes inflammatory mediators and adiponectin [62] that changes levels of the inflammatory secretome, including adipokine levels, which alter macrophage recruitment and impact CIMT progression indirectly [67]. Further, the type of hormone replacement (whether oral or transdermal) has been shown to slow down the change in pericardial fat accumulation after 2 years of treatment, highlighting a role in estradiol in mediating changes in fat composition [66].

### 4.7. Inflammatory Changes with Menopause

The onset of menopause is associated with increases in pro-inflammatory markers such as c-reactive protein, cytokine production (e.g., tumour necrosis factor α (TNF-α)), interleukins (e.g IL-1 and IL-6), and interferon-γ (IFN-γ) [68]. Levels of inflammatory markers have been shown to reduce with hormone therapy, suggesting a potential influence of estrogen on the immune system. Among the several inflammatory markers, CRP has been identified as one of the strongest independent risk factors for cardiovascular events and mortality among postmenopausal women [69]. The released cytokines not only amplify more cytokine production but also increase the permeability of endothelium and facilitate the process of atherogenesis [70]. As an example, cytokine-induced increased activity of the redox-sensitive transcription factor NF-κβ could drive the expression of matrix metalloproteinases and promote plaque rupture [70]. Cytokines also amplify the production of heat shock proteins (HSP) [70]. Elevated HSP levels have been found to predict progression of atherosclerosis in hypertensive patients and HSP also markedly enhances IL-6 production by macrophages, leading to a cycle of inflammation [70]. In addition to the effect on atherosclerosis, elevated inflammatory cytokines (e.g., TNF-α) may stimulate skeletal muscle proteolysis and induce skeletal muscle insulin resistance by negatively regulating insulin signal transduction to glucose uptake [71]. Dysregulation of skeletal muscle insulin sensitivity could predispose women to metabolic complications after menopause and insulin resistance is a risk factor for CVD [71].

Estrogen exerts its protective effect by modulating the gene expression of transcription factors following interaction with estrogen receptors or by indirectly upregulating antioxidant gene expression, which could increase endothelial nitric oxide synthase activity [72]. Nitric oxide (NO) plays an important role in vascular relaxation [72]. Increased NO production has been observed with estrogen administration to endothelial cells in vitro [72]. Estrogen also interacts with ER via a nongenomic pathway, resulting in inhibition of NF-κβ activation which leads to upregulation of IL-1, IL-6 and TNF-α [73]. Atherosclerosis could also be caused by abnormal monocyte adhesion and transmigration in the vasculature. In cell studies, supraphysiological doses of estrogen inhibited monocytic cell adhesion to endothelial cells [74]. Similarly, in young ovariectomised rabbit models, estrogen replacement at physiological concentrations increased monocyte adhesion and subendothelial migration [73]. Hormone replacement therapy in postmenopausal women has also been shown to decrease VCAM-1 levels and, as such, estrogen blocks monocyte adhesion and transmigration in the vasculature and could protect against atherosclerosis [75]. The effect of estradiol replacement could extend beyond the vascular protective effects. Torres MJ et al. showed that estradiol could affect skeletal muscle by binding to mitochondria independent of ERα and the direct binding of estradiol to mitochondria could modulate membrane fluidity and bioenergetic function, resulting in regulation of cellular redox homeostasis and maintenance of insulin sensitivity in ovariectomised rodents [76]. Thus, estradiol replacement could have benefits in protection against atherosclerosis and improvement of insulin sensitivity.

## 5. Treatment Considerations for Prevention of Ischemic Stroke in Women

### 5.1. Anti-Hypertensive Agents

Due to the differences in drug transporters and enzymes involved in clearance, women and men are likely to exhibit different pharmacokinetic profiles with anti-hypertensive medications [77]. Clearance of calcium-channel blockers (e.g., verapamil and amlodipine) are faster in females compared to men due to higher activity of CYP3A4 and lower activity of plasma-glycoprotein [77], but data on differences in outcomes have not been reported in major trials. Women are also more prone to adverse effects associated with the use of CYP2D6-dependent beta-blockers (e.g., metroprolol) compared to men [78]. Women are also more likely to report edema associated with use of calcium-channel blockers [77] and women also tend to have a 3-fold increase in angiotensin-converting enzyme inhibitor-related cough [77]. As a result of these reported events, women are less likely to receive ACE-inhibitors after stroke or for blood pressure management.

### 5.2. Lipid Lowering Therapy

Compared to men, women of reproductive age are often less likely to be started on high-intensity statins for primary stroke prevention [79]. This is because low-density lipoprotein levels tend to remain lower in women compared to men and often do not rise until after menopause [80]. Furthermore, there are insufficient data to indicate benefits from statin therapy for primary prevention of major coronary events and stroke in women. Statin use has also been associated with fetal anomalies and treatment of dyslipidemia is not recommended during pregnancy unless women have a significant cardiovascular risk factor, such as homozygous familial hypercholesterolaemia, or a prior history of cardiovascular disease. Notably, evidence suggesting fetal anomalies with high-intensity statin were only reported in animal studies. High-quality data in humans is lacking and a recent observational study has not shown any increased fetal anomalies in pregnant women treated with statin [81].

### 5.3. Anticoagulation for Atrial Fibrillation

Anticoagulants reduce thromboembolic events in AF and have been shown to have a greater effect in females compared to men [82]. Conversely, rates of thromboembolism in AF patients not taking anticoagulation were also much higher in women compared to men (3.5% per year for women compared to 1.8% per year for men, adjusted RR 1.6, 95% CI 1.3–1.9) [83]. Prior to the era of non-vitamin K oral anticoagulants (NOAC), there was a general perception that women could have increased bleeding complications with warfarin use due to the differences in pharmacokinetic clearance of the medication and the challenge in maintaining the INR within therapeutic range [84,85]. However, the introduction of NOACs appears to have reduced these sex-related disparities [86]. A single-centre prospective observational study involving 806 patients with AF showed that bleeding events were comparable between men and women treated with NOAC, and both genders benefitted from NOAC equally despite different background characteristics [86,87]. Furthermore, a meta-analysis comparing NOACs and warfarin reported a higher risk of thromboembolism among women treated with warfarin compared to men whereas no such sex-based differences were observed in the NOAC group. Bleeding risks were also similar for both sexes, regardless of whether patients were treated with NOACs or warfarin [88]. However, a real-world study by Ferroni et al. reported a higher risk of gastrointestinal bleeding among women on NOACs compared to men, but paradoxically, women had a lower bleeding risk than men when treated with warfarin [89]. These findings highlight the need for further research to better understand sex-specific responses to different anticoagulant therapies and to optimize personalized treatment strategies.

### 5.4. Left Atrial Appendage Occlusion (LAAO)

Left atrial appendage occlusion (LAAO) serves as an alternative therapy to anticoagulation for reducing thromboembolism risk in patients with nonvalvular AF [90]. By excluding the left atrial appendage from the systemic circulation, LAAO effectively reduces ischemic stroke risk by preventing thrombus formation. This procedure is particularly suitable for AF patients who are not ideal candidates for anticoagulation [91]. In a real-world population of older individuals with AF, women treated with LAAO had a reduced risk of stroke and systemic embolism compared to those treated with anticoagulation (HR 0.655 [95% CI, 0.555–0.772]). [92]. However, a study by Darden et al. found that compared to men, women were at a higher risk of adverse events (odds ratio (OR), 1.63; 95% CI, 1.49–1.77) after LAAO, such as pericardial effusions requiring drainage or major bleeding [93]. These findings highlight the need for further research to assess the safety of LAAO procedures and to develop strategies aimed at reducing procedure-related adverse effects in women.

## 6. Recanalization Therapy for Acute Ischemic Stroke in Women

### 6.1. Intravenous Thrombolysis

Intravenous thrombolysis (IVT) with alteplase or tenecteplase is the current approved treatment for acute IS with the aim of re-establishing cerebral perfusion. A meta-analysis of 18 published studies reported that women had significantly lower odds of receiving intravenous thrombolysis treatment than men (OR 0.70 (95% CI 0.55–0.88) [94]. Several factors may contribute to this disparity, including the concurrent use of anticoagulants, atypical stroke presentations in women that can lead to delayed diagnoses, and out-of-hospital delays in acute treatment [95]. Nevertheless, pooled analyses from randomized trials indicate that women derive significantly greater benefits from intravenous thrombolysis treatment compared to men [94,96]. While women treated with IVT show comparable functional outcomes to their male counterparts, untreated women experience worse outcomes than untreated men [96]. Higher or similar recanalization rates have been reported in women compared to men in the first 72 h following IVT [97,98]. Further research is essential to elucidate the underlying reasons for the lower utilization of IVT in women, as well as to explore the reasons for its seemingly enhanced benefits in this population. Importantly, there is a pressing need for strategies aimed at increasing the utilization of IVT among women to ensure they receive optimal acute IS care.

Intravenous thrombolysis is indicated for the treatment of acute ischemic stroke within 4.5 h of onset with disabling neurological deficits [99]. Recently, evidence has emerged supporting its use beyond the 4.5-h window for selected patients guided by perfusion neuroimaging. In the Extending the Time for Thrombolysis in Emergency Neurological Deficit (EXTEND) trial, Ma et al. demonstrated that administering alteplase between 4.5 and 9 h after stroke onset resulted in a higher percentage of patients with no or minor neurologic deficits than the use of placebo in patients with an ischemic core of less than 70 mL and a salvageable perfusion lesion [100]. Notably, there were more cases of symptomatic cerebral hemorrhage in the alteplase group than in the placebo group [99]. A pooled analysis of individual patient data from three clinical trials further supports the use of alteplase in patients with favorable perfusion imaging within 4.5 to 9 h after stroke onset, as well as in those with wake-up strokes, showing an adjusted odds ratio of 1.86 (95% CI 1.15–2.99) for improved functional outcomes at 3 months compared to placebo [101]. This is comparable to the previously reported odds ratio of 1.26 (95% CI 1.05–1.51) for patients treated within 3 to 4.5 h [101]. Based on these findings, guidelines now recommend thrombolysis with alteplase for patients presenting 4.5–9 h after stroke onset with neuroimaging showing core–perfusion mismatch and when mechanical thrombectomy is not indicated [102]. However, there remains a gap in research on the potential sex differences in the efficacy and risks of thrombolysis in this extended treatment window, underscoring the need to evaluate the utility of extended window thrombolysis specifically in women. 

### 6.2. Endovascular Thrombectomy

Endovascular thrombectomy (EVT) has become the standard of care for both anterior circulation and basilar artery large vessel occlusion acute IS. A recent meta-analysis found no significant sex differences in the utilization of EVT or in functional outcomes following EVT in patients with acute IS and large vessel occlusion. In a propensity-matched cohort from the SELECT study [103], women had discharge outcomes comparable to those of men following EVT. However, the improvement in outcomes from discharge to 90 days was significantly less pronounced in women, indicating that post-discharge factors may play a critical role in their recovery [104].

## 7. Stroke Outcomes in Women

Women have been reported to experience poorer functional outcomes and health-related quality of life following a stroke. According to Gall et al., women are generally older, in poorer health, and are twice as likely to live alone with limited social networks at the time of their stroke [105]. Living alone may also lead to delayed presentation to the hospital [106]. The combination of older age and poorer pre-stroke health contributes significantly to worse outcomes in women. Depression is common following stroke- and post-stroke-related depression is two times more likely in women than in men [107]. Untreated depression can adversely affect recovery after a stroke and significantly impact quality of life, ultimately leading to poorer outcomes [105]. A study conducted over an 8-year time period in an Austrian stroke unit showed that despite similar acute stroke care and neurorehabilitation, women had 1.26 odds of worse functional outcome at the 3-month follow up compared to men [108]. This finding is consistent with a report derived from the National Health Interview Survey where the authors found women to be more likely to report difficulty in performing functional activities such as walking, climbing 1 step, standing or stooping compared to men [109]. Given the poorer outcomes observed in women following a stroke, further research is essential to explore how clinical practice can enhance quality of care or modify pre-stroke risk factors for women. Additionally, large population-based studies are crucial to evaluate the cost-effectiveness of gender-specific stroke care, ensuring improved outcomes for women.

## 8. Conclusions

Sex is an important biological determinant of ischemic stroke. Although there is increasing recognition of female-specific risk factors (Table 1) and the complex interaction between sex, age, and cerebrovascular disease, significant research gaps remain. There is an urgent need for strategies to improve stroke prediction, prevention, and post-stroke care for women, empowering them to maintain independence and quality of life. With the rising burden of stroke among women, advancing research to better understand and address sex-related disparities in stroke outcomes is essential to pave the way for personalized, gender-sensitive stroke care.

## Figures and Tables

**Table 1:** Showing the gender-specific differences in stroke risk factors, treatment precautions, and prognosis of stroke. N/A: not applicable.

Risk Factors	
Hypertension	Blood pressure rises more sharply in women with age, with differences evident from the third decade. Stage 2 hypertension increases stroke risk by 30% in women compared to men. Treatment response rates for hypertension vary amongst men and women.
Diabetes Mellitus	The prevalence of diabetes is lower in pre-menopausal women compared to men; however, the risk of developing diabetes increases significantly after menopause. Higher risk of ischemic stroke in women than men, especially for type 1 diabetes. History of gestational diabetes increases ischemic stroke risk.
Atrial Fibrillation	Higher incidence in men, but prevalence increases in women at later ages. Female sex is a prognostic factor for stroke in AF patients, especially with two or more other risk factors. Women with AF are often undertreated with anticoagulation compared to men.
Female-specific Risk Factors	Age of menarche and age of menopause. Hypertensive disorders of pregnancy. Metabolic and inflammatory changes with menopause. Oral contraceptive use. Hormone replacement therapy.
Preventive Treatment	
Anti-hypertensive Agents	Women and men show different pharmacokinetic profiles for anti-hypertensive medications. Calcium-channel blockers are cleared faster in women with adverse effects more common (e.g., edema and ACE inhibitor-related cough).
Lipid-Lowering Therapy	Women of reproductive age are less likely to be prescribed high-intensity statins for primary stroke prevention due to lower LDL levels compared to men. Less data on statin benefits for the primary prevention of ischemic stroke in women. Statin treatment is not recommended during pregnancy unless significant cardiovascular risk.
Anticoagulation for AF	Anticoagulants reduce thromboembolic events in AF with greater effect observed in women. Higher thromboembolism rates in untreated women compared to men. Women could have increased bleeding complications with warfarin use due to the differences in pharmacokinetic clearance of the medication and the challenge of maintaining the INR within the therapeutic range. NOACs have reduced sex-related bleeding disparities.
Left Atrial Appendage Occlusion for AF	Women had a higher risk of post-procedural adverse events such as pericardial effusions requiring drainage or major bleeding.
Acute Treatment and Prognosis	
Intravenous Thrombolysis	Women had lower odds of receiving thrombolysis compared to men. Women derive greater benefits from thrombolysis and show comparable functional outcomes to men. Higher or similar rates of recanalization were achieved in women compared to men.
Endovascular Thrombectomy	No significant sex differences in EVT utilization or outcomes.
Prognosis and Outcomes	Women generally experience poorer functional outcomes and health-related quality of life after a stroke compared to men. Post-stroke depression is twice as likely in women than in men.

## Data Availability

No new data were created or analyzed in this study.
